# Tribute to Philippe Gasque (1966–2024): From Complement Biology to Alphavirus Pathogenesis and Chikungunya Research

**DOI:** 10.3390/v17111519

**Published:** 2025-11-20

**Authors:** Wildriss Viranaïcken, Gregorie Lebeau, Hoarau Jean-Jacques, Pascale Krejbich-Trotot

**Affiliations:** 1 UMR 1188 Diabète Athérothrombose Réunion Océan Indien (DéTROI), INSERM, Université de La Réunion, 97410 Saint-Pierre, France; 2 Department of Infectious Diseases, School of Immunology and Microbial Sciences, King’s College London, London SE19RT, UK; gregorie.lebeau@kcl.ac.uk; 3 Unité de Recherche en Pharmaco-Immunologie (UR-EPI), Université de La Réunion, 97490 Saint-Denis, France; jean-jacques.hoarau@univ-reunion.fr (H.J.-J.); pascale.krejbich@univ-reunion.fr (P.K.-T.)

The passing of Professor Philippe Gasque on 11 July 2024 marked the loss of a prominent figure in immunology and infectious disease research. Over three decades, his career bridged fundamental studies of the complement system with translational research on viral infections, particularly arthritogenic alphaviruses such as chikungunya virus (CHIKV). His unique expertise, combining neuroimmunology, complement biology, and viral pathogenesis, positioned him as a leading voice in the field. This article reviews his scientific journey and highlights his major contributions, with a focus on his pioneering insights into chikungunya pathophysiology.

**Rewriting the Rules of Complement Immunity**

As young student, Philippe Gasque, originating from the small French overseas island of La Réunion, located in the middle of the Indian Ocean, moved to mainland France to pursue his graduate education. He obtained his PhD in Science from the University of Rouen, where he conducted his doctoral research under the supervision of Dr Marc Fontaine. Following the completion of his PhD, he joined Prof Paul Morgan’s research group based at the College of Medicine of the University of Wales (Cardiff, UK) in 1993. During these two research periods, Gasque’s early scientific career was dedicated to the complement system. He focused on a core belief in immunology that was considered settled science, and in challenging it, he laid the foundation for his life’s work.


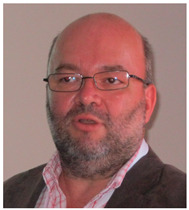


For decades, the scientific consensus was that complement proteins, the foot soldiers of this immune system branch, were produced almost exclusively in the liver. Gasque’s landmark studies in the early 1990s methodically overturned this dogma, revealing a far more intricate and localized system. He was among the first to prove that cells within the Central Nervous System (CNS), including astrocytes, microglia, and even neurons, could produce their own complement proteins. The brain, it turned out, had its own localized defense factory. More specifically, during his PhD, he demonstrated the expression of complement system components by astrocytes. Later, during his postdoctoral fellowship as an International Travelling Fellow of the Wellcome Trust, he showed that not only the terminal components of the complement system are expressed in these cells, but also several complement receptors, notably those involved in cell activation and chemoattraction (CR2, C5aR, C3aR). He discovered that pro-inflammatory signals, known as cytokines, were the trigger that activated this local complement production, directly linking the process of neuroinflammation to the complement cascade.

In 1996, supported by a four-year Career Development Award from the Medical Research Council (UKRI MRC), he demonstrated both in vivo and in vitro that, beyond the complement system, other cytotoxic pathways are also active in the brain, particularly under pathological conditions such as Alzheimer’s disease, stroke, and multiple sclerosis. During this period, he was the first to describe that perforin, a protein initially thought to be produced by cytotoxic T cells and natural killer cells, is also expressed by fetal astrocytes. In the adult brain, however, astrocytic perforin expression was found to be restricted to reactive astrocytes located in and around areas of inflammation, within both white and gray matter, in multiple sclerosis and other neurodegenerative conditions. From 1998, he became the head of the Brain Inflammation and Immunity Group (BIIG) and was appointed Senior Lecturer with a Senior Fellowship from the MRC at the Department of Medical Biochemistry and Immunology at Cardiff University. BIIG quickly established itself as a leading center in the field of neuroimmunology. During this period, he focused on the crosstalk between the complement system and neurodegenerative as well as neuroinflammatory diseases. He paid particular attention to the regulation of the innate immune system in the CNS and developed the concept of Neuro-immune-Regulators (NIREGs)**,** a group of proteins expressed in the central nervous system that modulate complement activation and immune responses. In collaboration with colleagues such as the neuropathologist Dr James Neal, he proposed that these proteins help maintain neural homeostasis by regulating inflammation and protecting neural tissues from excessive cytotoxicity, thereby playing a crucial role in both neuroprotection and the pathogenesis of neurodegenerative and neuroinflammatory disorders.

His work revealed the complement system’s dual nature in the brain: it could be a guardian, clearing threats, but its over-activation could also drive the very damage seen in neurodegenerative diseases and brain injury. By proving that complement was not just a systemic, liver-based system, Gasque helped establish a vital new concept for science: tissue-specific complement biology. This insight fundamentally changed how researchers understood neuroimmunology and inflammation, revealing that battles between immunity and disease were being fought locally in tissues all over the body. While Gasque was mapping the brain’s hidden immune defenses, an entirely different kind of threat was gathering force, one that would soon bring his theoretical work into sharp, practical focus.

**A Career’s Turning Point: The 2005–2006 Chikungunya Epidemic in The Indian Ocean**

In 2005, at the request of several local academics and to be closer to his parents, Philippe Gasque decided to return to his native island and obtained a position as a full professor at the University of La Réunion. He initially developed a research program focused on adipose tissue as a key player in innate immunity in diseases, mainly in type 2 diabetes, ischemia, and sepsis. But it is the major outbreak of chikungunya virus that occurred in 2005–2006 that profoundly reshaped Prof Gasque’s research focus. At that time, he contributed to the establishment of a cutting-edge research platform (CYROI) by leading collaborative research projects. He also participated in the governance of the CYROI public interest group for several years. This platform became the hotspot of research on the island notably hosting a center dedicated to the monitoring of emerging diseases in the Indian Ocean (CRVOI). This also became a successful attempt to place island regions at the heart of tropical virology research. During that period, he also founded a research group in infectious diseases and immunity (GRI), with the ambitious goal of bringing together scientists and hospital practitioners facing previously undescribed clinical forms of the disease, in a pioneering “from bench to bedside” translational approach. He thus succeeded in federating a wide range of expertise and led this team until 2015.

As a scientist based on the island, he was not a distant observer but a key figure on the frontline, uniquely positioned to document and decipher the outbreak’s devastating consequences. He devoted all his energy to structuring and coordinating a robust, multidisciplinary scientific response to the crisis in this small tropical region, which, in the globalized era we have been experiencing since the 2000s, has become a hotspot for the emergence of arboviruses and a regional hub at risk of importation (from Comoros, Madagascar, Mauritius and Mayotte). He succeeded in joining together the forces available and developing the required infrastructure to carry out local research focused on experimental virology and the subtle mechanisms of the host response to the pathogen. Gasque’s team sounded the alarm, publishing critical reports on the previously underappreciated severity of the disease. They documented life-threatening cases requiring intensive care, including hepatic failure, cardiovascular decompensation, acute epidermolysis and severe neurological syndromes such as encephalitis and Guillain–Barré-like disorders. For Gasque, neuroimmunologist by training, these cases of encephalitis were not just tragic clinical outcomes; they were a direct challenge that echoed with his earliest research into the brain’s private immune system. The epidemic provided a wealth of clinical data, but for a scientist like Gasque, it also raised a series of deeper, more perplexing biological questions. The paradox that captivated Gasque was one of immunological civil war: the very systems designed to protect the host were being co-opted by the virus to sustain a chronic, painful, and destructive inflammation. He dedicated his later career to solving this central mystery of chikungunya.

One of his most important discoveries was identifying where the chikungunya virus (CHIKV) could hide from the immune system. His widely cited work described the phenomenon of persistent chronic inflammation despite a robust host immune response, a central concept in understanding post-viral syndromes. He demonstrated that even after the initial infection was cleared from the bloodstream, the virus could persist in cellular reservoirs, hiding spots primarily in immune cells called macrophages (the body’s ‘clean-up crew’ that engulfs cellular debris and pathogens) and in the synovial tissues (the delicate lining of the joints). These viral sanctuaries constantly fueled the chronic, arthritis-like joint pain that became the disease’s hallmark. He also developed collaborative studies with clinicians to better understand the pathophysiology of the disease, particularly focusing on elucidating the empirical use of methotrexate in the management of post-chikungunya chronic pain and persistence.

Gasque’s group also uncovered a clever strategy the virus uses to propagate, to diversify its tropism and find a way to persist. They found that CHIKV induces apoptosis in the infected cells. The virus uses the body’s own “cleanup” process as a Trojan horse: the dying cell breaks apart into small packages called apoptotic bodies, which still contain the virus. Not only can neighboring cells absorb these packages, becoming infected themselves, but professional phagocytes, by performing their expected non-phlogistic cleaning of virus-laden apoptotic residues, become privileged niches that ensure the long-term persistence of CHIKV in their human host. This mechanism allows the virus to spread efficiently while evading immune detection and amplifying tissue damage. His work on the virus’s tactics set the stage for the final, crucial step in his scientific journey: connecting these new viral insights back to his foundational research. A key finding highlighted by Gasque’s team also concerned the way in which CHIKV manipulates diverse host cell stress pathways and how that relates to viral replication and disease severity. Their study demonstrating CHIKV’s ability to use autophagy to enhance its replication paved the way for understanding the dual role of this cellular response. Indeed, while adaptive autophagy following infection could help get rid of viral capsids, thereby limiting the virus egress, the human NDP52 receptor, unlike its murine ortholog, would promote viral replication by interacting with the nonstructural protein nsP2 of CHIKV. This revealed species-specific characteristics, highlighting new limitations of the murine model to study CHIKV. Gasque’s team contributions were decisive because they moved the field beyond descriptive clinical observations into mechanistic cellular and molecular insights. These studies established the GRI as a leading research center for emerging infectious diseases in the Indian Ocean region and contributed to a broader understanding of host–pathogen interactions in arboviral infections. Recognition of his dedicated work came with the organization of the 2nd International Conference on Infections of the Nervous System (IANIS) meeting, which he hosted in November 2010 on the island of La Réunion. The meeting focused on chikungunya and infections affecting the central nervous system, bringing together leading experts from around the world in these fields. The conference was preceded by a joint ARC-WERC IBRO School session on “Pathogenic and neuroprotective mechanisms in CNS infections prevalent in developing countries”, open to early-career researchers, offering them a unique opportunity to learn about the pathogenesis and important gaps in knowledge of some of the most prevalent diseases of the nervous system, which otherwise are rarely addressed in neuroscience courses. As a major contributor to the understanding of chikungunya pathophysiology, he was subsequently involved in establishing the Integrated Chikungunya Research (ICRES) consortium from 2010 to 2014. This collaborative project, supported by the European Union under the Health Cooperation Work Programme of the 7th Framework Programme (FP7), brought together leading international specialists in chikungunya research from UK, Sweden, Estonia, Finland, Germany, France, Singapore and Malaysia, including but not limited to, Marc Lecuit, Pierre Roques, Peter Liljeström, Andres Merits, Lisa NG and John Fazakerley. This coordinated research, conducted within and beyond the EU, has contributed to capacity building and the production of knowledge necessary to improve the surveillance, diagnosis and understanding of the pathological processes, as well as to the development of pre-clinical therapeutic candidates for the treatment and prevention of CHIKV.

This tribute gains even greater significance twenty years later, in the context of the recent chikungunya outbreak on the island in 2025, an event that Prof Gasque had long foreseen, warning for years that the progressive increase in the proportion of immunologically naive individuals within the population would inevitably heighten the risk of resurgence. In line with the paradigm, he has long defended regarding immune status, in the face of the current 2025 crisis and the rising number of cases, he would remind us that we have once again reached the level of coverage required for group protection until the next immune burst. Despite the apparent end of the epidemics, he insisted, against all odds, on maintaining active chikungunya research after 2015, anticipating a possible re-emergence. As he often emphasized, an epidemic represents only a moment in the virus’s pathophysiological activity, but its medium- and long-term consequences should compel us to sustain research on the biology of the virus, in preparation for future re-emergence driven by the cyclical loss of herd immunity and to pave the way for a deeper understanding of the mechanisms underlying post-chikungunya pain and the identification of new therapeutic targets.

**Connecting the Dots**

Even he focused much of the work on the biology of the chikungunya virus and alphaviruses, he continued to emphasize host–pathogen interactions, with a particular focus on the recognition of damage-associated molecular patterns (DAMPs) and pathogen-associated molecular patterns (PAMPs) in relation to the complement system, a research area to which he was deeply committed, inspired by the seminal work and paradigms of the renowned scientist Charles Janeway, whom he greatly admired. Gasque’s studies reinforce the hypothesis that damage-associated molecular patterns (DAMPs), such as HMGB1 or HSP60, do not exert an intrinsic pro-inflammatory activity per se. Rather, their immunostimulatory potential appears to depend on specific contextual factors, including their physical association with PAMPs or structural and post-translational modifications that distinguish them from their homeostatic, basal counterparts. Such findings further support the discontinuity theory of immune activation, which suggests that the immune system primarily responds to abrupt molecular or contextual changes rather than to the mere presence of self- or non-self-molecules.

For many years, he has expressed a deep fascination with mesenchymal stem cells (MSCs), consistently emphasizing the importance of neural crest–derived cells in orchestrating immune responses. Since 2015, his work has included analyses of MSC involvement in post-Chikungunya arthritis and in sepsis. More recently, he has opened a new line of investigation exploring the role of MSCs in cancer. Across these studies, Gasque’s work highlights that MSCs are not only passive “repair” cells; rather, they can be activated by danger signals or PAMPs, modulate immunity toward either activation or regulation, and even be targeted or exploited by pathogens, particularly viruses. The concept of MSCs serving as viral reservoirs or immune-privileged niches has been particularly emphasized. This has major implications for understanding viral persistence within specific tissues (e.g., joints, synovium), as well as for the design of therapeutic strategies involving MSCs, which must consider the potential risk that these cells might harbor or be functionally altered by infection. In infectious or inflammatory contexts, MSCs can acquire an immunoregulatory or inhibitory phenotype rather than a pro-inflammatory one, thereby potentially promoting infection chronicity or immune evasion. He addressed these concepts in several reviews that have since become key references in the field. Besides this, the three parallel streams of Gasque’s career finally converged at the end. The local immune factory he had discovered in the brain two decades earlier was not merely a theoretical curiosity; he now had evidence that it constituted a central battleground in the fight against cancer. In recent years, he proposed the use of recombinant chikungunya viruses expressing complement molecules with anaphylatoxin activity for the treatment of poor-prognosis cancers, the so-called cold tumors. The oncolytic properties of the virus, combined with the immunoregulatory roles of complement components, could, according to his hypothesis, enable a synergistic action involving the induction of apoptosis and the recruitment and activation of immune effector cells within the tumor. Considering the tropism, he had demonstrated in his previous work, he suggested preferentially targeting glioblastomas (a poor-prognosis tumor), which display high permissiveness to this virus, along with the ability of complement molecules to exacerbate inflammation in CNS.

**An Enduring Legacy**

Professor Gasque’s work has left a lasting influence across three interrelated domains of science and medicine. His pioneering contributions to complement biology, particularly the discovery of local complement production, continue to inform a wide range of research areas, from the study of neurodegenerative disorders such as Alzheimer’s disease to the complex immunological challenges of organ transplantation. In the field of viral pathogenesis, his conceptual framework describing how viruses can hijack host cell responses and trigger post-viral chronic syndromes has proven highly relevant beyond Chikungunya infection, providing a valuable model for understanding conditions such as long COVID. Finally, through his commitment to addressing the Chikungunya epidemic in La Réunion, Professor Gasque exemplified the ideal of a translational immunologist, a scientist who bridges fundamental immunology with clinical and public health realities, transforming local crises into opportunities for global scientific insight.

Professor Philippe Gasque embodied the ideal of a scientist: a researcher who uses deep molecular insights to solve pressing clinical problems. From the intricate biology of complement proteins to the devastating pathogenesis of chikungunya, his work consistently illuminated a central truth of immunology: that our immune system can be both a guardian and a driver of disease. While his passing is a profound loss to the scientific community, his discoveries and the researchers he trained ensure the bridge he built between fundamental immunology and human disease will be traveled by scientists for generations to come. Alongside this intense research activity, he has continuously worked to strengthen research and education at the University of La Réunion. He contributed to the establishment of the University Hospital (CHU) in 2012 together with Professor Pascale Guiraud, Dean of the Faculty of Health, and to the creation of a Research Master’s program in Biological and Health Sciences in 2010. In this part of this life, Gasque was not a simple professor, but someone who opened doors to the exciting unknowns that science invites us to explore. Whether he was discussing a groundbreaking theory or guiding students through their very first research project, he brought the same spark, the same energy, the same unshakeable belief that discovery is one of humanity’s greatest endeavors. With Prof Gasque, complex ideas became not only accessible, but alive, and beyond his publications, he mentored numerous students and built collaborations across disciplines. His intellectual rigor, combined with his commitment to the people of La Réunion and the global scientific community, will continue to inspire researchers.

